# Four *Pristionchus* species associated with two mass-occurring *Parafontaria laminata* populations

**DOI:** 10.21307/jofnem-2020-115

**Published:** 2020-11-30

**Authors:** Natsumi Kanzaki, Minami Ozawa, Yuko Ota, Yousuke Degawa

**Affiliations:** 1 Kansai Research Center, Forestry and Forest Products Research Institute, 68 Nagaikyutaroh, Momoyama, Fushimi, Kyoto 612-0855, Japan; 2 College of Bioresource Sciences, Nihon University, Fujisawa, Kanagawa 252-0880, Japan; 3 Sugadaira Research Station, Mountain Science Center, University of Tsukuba, 1278-294 Sugadairakogen, Ueda, Nagano 386-2204, Japan

**Keywords:** Ecology, Genotyping, Millipede, *Parafontaria laminata*, Phorecy, *Pristionchus*

## Abstract

Phoretic nematodes associated with two mass-occurring populations of the millipede *Parafontaria laminata* were examined, focusing on *Pristionchus* spp. The nematodes that propagated on dissected millipedes were genotyped using the D2-D3 expansion segments of the 28S ribosomal RNA gene. Four *Pristionchus* spp. were detected: *P*. *degawai*, *P*. *laevicollis*, *P*. *fukushimae*, and *P*. *entomophagus*. Of the four, *P*. *degawai* dominated and it was isolated from more than 90% of the millipedes examined. The haplotypes of partial sequences of mitochondrial cytochrome oxidase subunit I examined for *Pristionchus* spp. and *P*. *degawai* showed high haplotype diversity.

The genus *Pristionchus* (Kreis, 1932) is a satellite model system in many different fields of biology (Sommer, 2015). The flagship species *P*. *pacificus* (Sommer et al., 1996) is used to study phenotypic plasticity, kin recognition, and chemical biology (e.g., Ragsdale et al., 2013a, 2013b; Lightfoot et al., 2019), and its congeners show high biological and physiological diversity (e.g., fig-associated species) and provide comparative information on genome-level diversification and speciation (e.g., Rödelsperger et al., 2014; Susoy et al., 2016).

Therefore, dense taxon sampling of the genus has been conducted (e.g., Herrmann et al., 2006; Mayer et al., 2007; Kanzaki et al., 2012a), but the diversity of the genus is far from saturated (Ragsdale et al., 2015). The genus is distributed widely, i.e., at least one species including undescribed ones have been isolated from all major continents (Ragsdale et al., 2015; Wang et al., 2015; Susoy et al., 2016), and in the previous studies, the genus has been isolated from relatively nutrient-rich substrates in European countries, partially because the taxonomy of *Pristionchus* has not been conducted in other areas of the world (e.g., Herrmann et al., 2015; Ragsdale et al., 2015). However, recently, their close association with wide-ranged invertebrates, mostly insects, has been recognized (e.g., Herrmann et al., 2006; Kanzaki et al., 2012a, 2012b, 2013), and the isolation from these hosts, especially Scarabaeidae (Ragsdale et al., 2015), are increasing. In addition, recent surveys have found that many nominal and undescribed *Pristionchus* species are also associated with soil arthropods, such as millipedes (Kanzaki et al., 2016, 2018; Kanzaki unpubl. obs.).

Biologically, *Pristionchus* is phoretic and necromenic nematodes. The nematodes are isolated from their host/carrier insects as dauer ( = dormant and dispersal) stage, which can be reared on artificial media, suggesting phoretic association, but the worms also propagate on the carcass of their host/carrier insects utilizing the carcass as the substrate of their food bacteria (e.g., Rae et al., 2008; Cinkornpumin et al., 2014). Thus, the insects which can be utilized in multiple ways are very important for the nematodes, and the genus possibly provide an insight for analyzing evolution of feeding preference and host/carrier usage.

To isolate *Pristionchus* spp., phoretic/necromenic nematodes were examined in two mass-occurring populations of *Parafontaria laminata* in Nagano, Japan. The mass-occurring population of *P*. *laminata* is known as ‘train millipede’ covers the ground and even disturb the train service covering the railroad (Toyota et al., 2006), and it was previously considered an independent subspecies, ‘*P*. *l*. *armigera* (Verhoeff)’, but is now synonymized to the original subspecies (Tanabe, 2002). *Parafontaria laminata* is the only species undergoing mass-occurrence every 8 years in the collection area, and easily identified based on its mass-occurrence and general morphology (Fig. 1). Four species of *Pristionchus*, an *Oscheius* sp., and a hind gut parasite (Thelastomatidae) were isolated, and two *Pristionchus* spp. that were undescribed at the time were described taxonomically as *P*. *degawai* (Kanzaki et al., 2018) and *P*. *laevicollis* (Kanzaki et al., 2018) before this study (Kanzaki et al., 2018).

**Figure 1:**
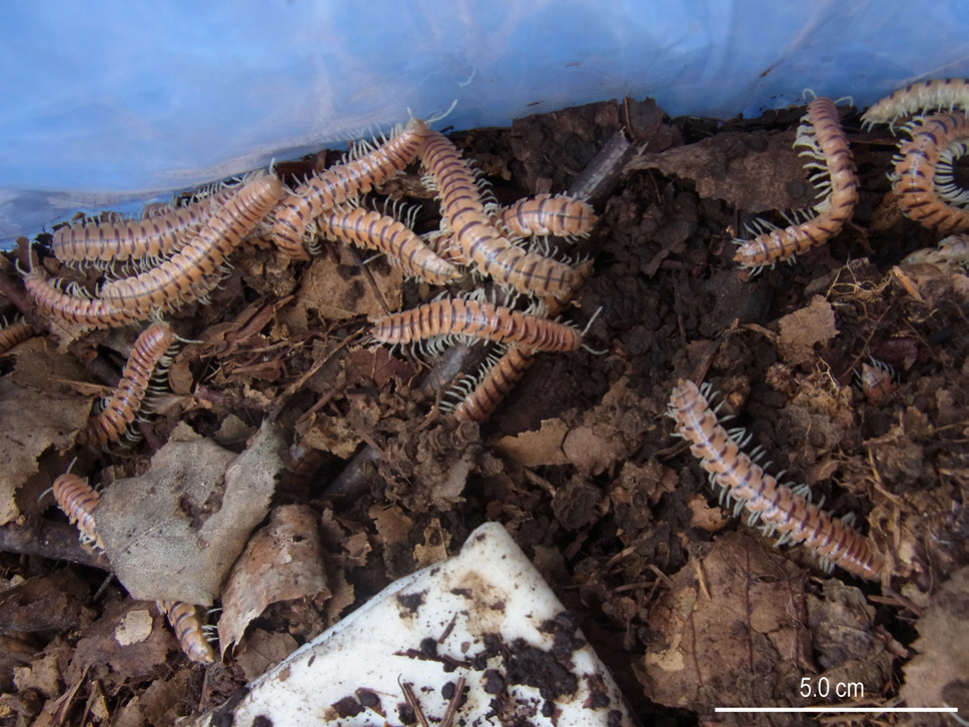
Millipedes (*Parafontaria laminata*) collected for this study.

The present study provides extensive information on the isolation of these four *Pristionchus* species.

## Materials and methods

### Collection of millipedes

Millipedes were collected manually in Koumi near Matsubara Lake (Mat) and Nobeyama (Nob), Minamimaki Village, Nagano, Japan in 2016. These sites are relatively cool mountain areas (> 1,100 m a.s.l.) in central Japan.

Collected millipedes were brought to the laboratory, and kept at 10 to 15°C until dissection.

### Nematode isolation, culture, and genotyping

First, millipedes (33 individuals from Mat and 38 from Nob) were individually dissected on water agar (2.0% agar in a *φ* = 90 mm Petri dish) and kept at ca. 20°C for 1 week. After the propagation of *Pristionchus* spp. was confirmed, 3 to 5 individual nematodes were hand-picked from each plate, transferred individually to nematode digestion buffer (Kikuchi et al., 2009; Tanaka et al., 2012), and genotyped based on the D2-D3 LSU sequence, as described in Ye et al. (2007). Three species of *Pristionchus* were recognized: *P*. *fukushimae* from Mat, *P*. *laevicollis* from Nob, and *P*. *degawai* from both localities; all examined millipedes harbored one or two *Pristionchus* species. *Pristionchus degawai* dominated in both millipede populations (Table 1). Here, two strains of *P*. *fukushimae*, one strain of *P*. *laevicollis*, and one strain of *P*. *degawai* were established. The last two were used as the type strains of each species (Kanzaki et al., 2018).

**Table 1.** Isolation of *Pristionchus* spp. in the preliminary study.

Matsubara Lake population			Nobeyama population		
Individual number	Gender	Nematode species	Individual number	Gender	Nematode species
Mat_1	M	*P. degawai*	Nob_1	F	*P. degawai*
Mat_2	F	*P. degawai*	Nob_2	M	*P. degawai*, *P. laevicollis*
Mat_3	M	*P. degawai*	Nob_3	M	*P. degawai*
Mat_4	F	*P. degawai*	Nob_4	M	*P. degawai*
Mat_5	F	*P. degawai*	Nob_5	M	*P. degawai*
Mat_6	M	*P. degawai*	Nob_6	M	*P. degawai*
Mat_7	F	*P. degawai*	Nob_7	F	*P. degawai*, *P. laevicollis*
Mat_8	F	*P. degawai*	Nob_8	F	*P. degawai*
Mat_9	F	*P. degawai*	Nob_9	M	*P. degawai*
Mat_10	F	*P. degawai*	Nob_10	F	*P. degawai*
Mat_11	F	*P. degawai*	Nob_11	F	*P. degawai*
Mat_12	F	*P. degawai*	Nob_12	F	*P. degawai*, *P. laevicollis*
Mat_13	F	*P. degawai*	Nob_13	F	*P. degawai*
Mat_14	F	*P. degawai*	Nob_14	M	*P. degawai*
Mat_15	F	*P. degawai*	Nob_15	F	*P. degawai*
Mat_16	F	*P. fukushimae*	Nob_16	F	*P. degawai*
Mat_17	F	*P. fukushimae*	Nob_17	M	*P. degawai*
Mat_18	M	*P. degawai*	Nob_18	M	*P. degawai*
Mat_19	F	*P. degawai*	Nob_19	F	*P. laevicollis*
Mat_20	F	*P. degawai*	Nob_20	F	*P. degawai*
Mat_21	M	*P. degawai*	Nob_21	M	*P. degawai*
Mat_22	M	*P. degawai*	Nob_22	M	*P. degawai*
Mat_23	F	*P. degawai*	Nob_23	F	*P. degawai*
Mat_24	F	*P. degawai*	Nob_24	M	*P. degawai*
Mat_25	F	*P. degawai*	Nob_25	F	*P. laevicollis*
Mat_26	F	*P. degawai*	Nob_26	F	*P. laevicollis*
Mat_27	F	*P. degawai*	Nob_27	F	*P. laevicollis*
Mat_28	M	*P. degawai*	Nob_28	M	*P. laevicollis*
Mat_29	M	*P. degawai*	Nob_29	M	*P. degawai*
Mat_30	F	*P. degawai*	Nob_30	M	*P. degawai*
Mat_31	M	*P. degawai*	Nob_31	F	*P. laevicollis*
Mat_32	F	*P. degawai*	Nob_32	M	*P. degawai*
Mat_33	F	*P. degawai*	Nob_33	M	*P. laevicollis*
			Nob_34	F	*P. laevicollis*
			Nob_35	F	*P. degawai*
			Nob_36	F	*P. degawai*
			Nob_37	F	*P. degawai*
			Nob_38	M	*P. degawai*

To examine more detailed associations, the partial sequence of the mitochondrial cytochrome oxidase subunit I (mtCOI) gene was determined for the established cultures. Millipedes (20 per population) were dissected on the agar plate, and kept at ca. 20°C, as described above. The agar plates were examined under a dissecting microscope (S8 Apo, Leica) daily for 2 weeks.

When any *Pristionchus* species was recognized, 5 to 10 first-found gravid females were transferred separately into Nematode Growth Medium (*φ* = 40 mm Petri dish) previously inoculated with the *Escherichia coli* OP50 strain. The propagated nematodes were subcultured and kept as laboratory strains. Using this procedure, 215 temporal strains were established. An individual nematode was hand-picked from each culture and genotyped based on the D2-D3 LSU sequence (as described above). In addition, all temporal strains and two strains of *P*. *fukushimae* established in the first isolation were genotyped based on the mtCOI sequence according to the methods in Kanzaki and Futai (2002), and the sequences were analyzed phylogenetically with the online version of PhyML (http://www.atgc-montpellier.fr/phyml/) in which the analytical parameters were selected automatically (Guindon et al., 2010).

## Results and discussion

### Species diversity

**Figure 2:**
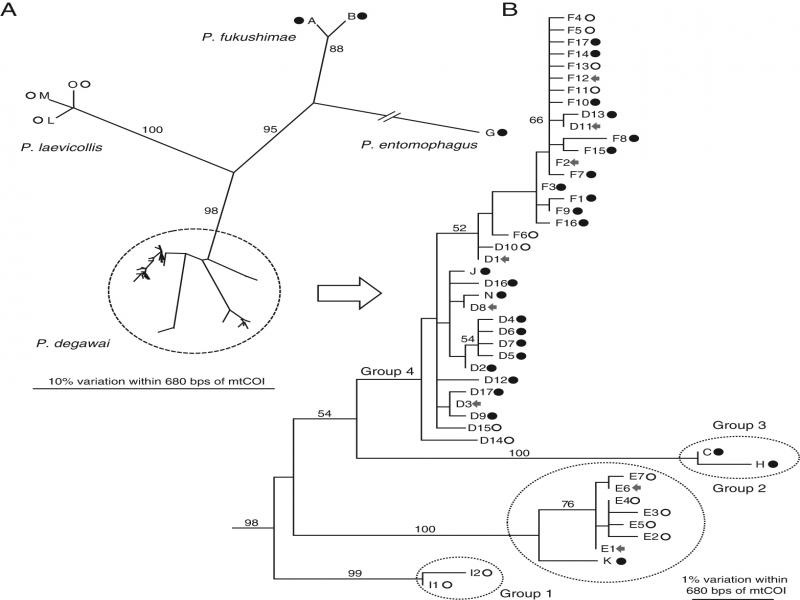
Phylogenetic relationships among the 54 haplotypes of four *Pristionchus* species found in this study. A: Unrooted tree showing the relationships among the four species; B: Phylogenetic relationships among the 47 genotypes of *P*. *degawai*. The Maximum Likelihood tree was inferred from partial sequences of the *mtCOI* gene. The GTR+G model was applied, and the parameters were as follows: lnL = –45,723.93825, freqA = 0.21, freqC = 0.10, freqG = 0.16, freqT = 0.43, R(a) = 3.9, R(b) = 100.0, R(c) = 15.8, R(d) = 14.0, R(e) = 100.0, R(f) = 1.0, and Shape = 0.12. Bootstrap values exceeding 50% are given on the appropriate clades. Some *P*. *fukushimae* strains did not amplify with the universal mtCOI primers (Kanzaki and Futai, 2002), probably because of a mutation in the primer region, and these strains were treated as a genotype (they do not appear in the tree). Symbols after haplotype codes indicate the haplotype found only from Nob (open circle), only from Mat (closed circle) and both Nob and Mat (arrow).

The 217 strains were separated into four species: *P*. *entomophagus* (one strain from Mat; one mtCOI haplotype), *P*. *fukushimae* (11 strains from Mat; three mtCOI haplotypes), *P*. *laevicollis* (three strains from Nob; three mtCOI haplotypes), and *P*. *degawai* (97 strains from Mat and 105 from Nob; 47 mtCOI haplotypes) (Fig. 2A and Table 2). The D2-D3 LSU sequences of these four species were identical to those deposited in GenBank (https://www.ncbi.nlm.nih.gov/genbank/?). The newly determined mtCOI sequences were deposited in GenBank with accession numbers LC589007-LC589060 (Table 3). In addition, an unidentified *Oscheius* sp. was isolated from all examined individual millipedes, although further study was not conducted for the species.

**Table 2.** Mitochondrial cytochrome oxidase subunit I haplotypes isolated in the extensive study.

Matsubara Lake population			Nobeyama population		
Individual number	Gender	Haplotype	Individual number	Gender	Nematode species
Mat101	M	D1, D3, D12, F8, N	Nob101	F	F11, L^a^, M^a^
Mat102	F	D1, D2, F17	Nob102	M	D8, F2, O^a^
Mat103	M	D3, D5, F1, F14, F15	Nob103	M	F2, D14
Mat104	F	D1, D8, D16	Nob104	M	D8, D14, F2
Mat105	F	D1, D12, F1	Nob105	M	D8, D14
Mat106	M	C, D1, D6, D16	Nob106	M	D8, F2, F6, E3, E6
Mat107	F	D3, D8, D17	Nob107	F	D8
Mat108	F	D1, D5, D6, F15, K	Nob108	F	D14, F12, F13
Mat109	F	D5, D8, D13, E1, E6, F1, K	Nob109	M	D1, E6, E7, L^a^
Mat110	F	D2, D3, D12, F1, K, X^a^	Nob110	F	F2, E4
Mat111	F	D1, D2, D11, E6, F2, F12	Nob111	F	D8, D14
Mat112	F	D1, D3, D7, F1, F8	Nob112	F	E1, I1
Mat113	F	D1, D3, D6, D12, F3	Nob113	F	D1, F4, F5, I2
Mat114	F	D3, D9, E6, F1, F2, F9, K, G^a^	Nob114	M	D1, F6, D8
Mat115	F	D1, D2, D3, D6, D13, F1, F7, F14	Nob115	F	F6
Mat116	F	D1, F1, F10	Nob116	F	D1, D8, D14
Mat117	F	D1, D6, D11, F8	Nob117	M	F2, F12
Mat118	M	D1, D2, D3, D4, D5, D6, D12	Nob118	M	D1, D11, E1, E5
Mat119	F	D1, D2, D3, F2, H	Nob119	F	D1, D3, D10, E2, J, O^a^
Mat120	F	D1, D2, D7, E6, F3	Nob120	F	D15, M^a^

**Notes:** ^a^Haplotype G is *P. entomophagus* (Mat114); L, M, O are *P. laevicollis* (Nob 101, 102, 109, 119 and 120); and X which was not amplified with universal primer set is *P. fukushimae* (Mat110). In addition to these strains, haplotypes A and B (*P. fukushimae* strains established in the first isolation) were included the phylogenetic analysis.

**Table 3.** GenBank accession numbers for the mtCOI haplotypes.

Type	Species	Accession number	Type	Species	Accession number
A	*P. fukushimae*	LC589007	E4	*P. degawai*	LC589034
B		LC589008	E5		LC589035
G	*P. entomophagus*	LC589009	E6		LC589036
L	*P. laevicollis*	LC589010	E7		LC589037
M		LC589011	F1		LC589038
O		LC589012	F2		LC589039
C	*P. degawai*	LC589013	F3		LC589040
D1		LC589014	F4		LC589041
D2		LC589015	F5		LC589042
D3		LC589016	F6		LC589043
D4		LC589017	F7		LC589044
D5		LC589018	F8		LC589045
D6		LC589019	F9		LC589046
D7		LC589020	F10		LC589047
D8		LC589021	F11		LC589048
D9		LC589022	F12		LC589049
D10		LC589023	F13		LC589050
D11		LC589024	F14		LC589051
D12		LC589025	F15		LC589052
D13		LC589026	F16		LC589053
D14		LC589027	F17		LC589054
D15		LC589028	H		LC589055
D16		LC589029	I1		LC589056
D17		LC589030	I2		LC589057
E1		LC589031	J		LC589058
E2		LC589032	K		LC589059
E3		LC589033	N		LC589060

Previous and present isolation records of *Pristionchus* spp. from millipedes are summarized in Figure 2. *Pristionchus entomophagus* is widely distributed in Europe (Kanzaki et al., 2014; Ragsdale et al., 2015), but this is the first report of the species in East Asia. *Pristionchus fukushimae* was originally described from stag beetle collected from northeast Japan (Fukushima Prefecture) (Ragsdale et al., 2013a, 2013b) and subsequently found from stag beetles and a soil sample in several relatively cool areas of Japan (Kanzaki, unpubl. obs.). Therefore, the species is considered widespread in the cool areas of Japan (and possibly other East Asian countries) mostly associated with decomposed plant materials and their related arthropods. *Pristionchus laevicollis* was previously isolated from *Aegus laevicollis subnitidus* in Nagoya (Kanzaki et al., 2018) and Kyoto (Kanzaki, unpubl. obs.), Japan, a relatively warm area, and might be distributed widely in Japan, although this remains unknown. So far, *P*. *degawai* has been isolated only from *P*. *laminata* (Kanzaki et al., 2018), and dominated the *Pristionchus* spp. associated with the millipede in this study.

**Figure 3:**
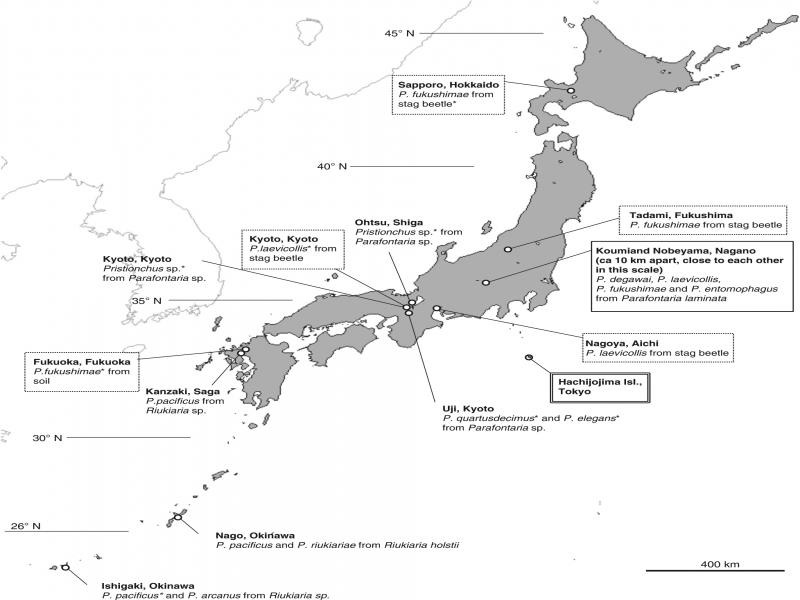
Previous isolation record of *Pristionchus* spp. associated with millipedes in Japan. The collection localities for the present study are suggested by solid line box, previous record of the species mentioned in the present study are suggested by dotted line box, and Hachijojima Isl. (Carta et al., 2018) is suggested by double line box. N. Kanzaki’s unpubl. obs. is indicated with an asterisk.

Considering millipedes’ bionomics, i.e., feeding on decomposing plant materials and inhabiting soil (Toyota et al., 2006), their habitat partially overlap stag beetles (Tanahashi et al., 2009), and it is not surprising that these hosts share the same nematode species. In addition, relatively wide host (carrier) range of *Pristionchus* spp. (*P. fukushimae*, *P. laevicollis*, and *P. entomophagus*) was confirmed. *Pristionchus* spp., except for fig associates, is not only phoretic, but also necromenic species. Thus, considering their host as a substrate, host specificity is not necessary to become strict. Contrastingly, many species inhabiting newly-dead wood, e.g., *Micoletzkya* (diplogastrid bacteria feeder) and *Bursaphelenchus* (aphelenchoidid fungal feeder), have relatively high host/carrier specificity (Susoy and Herrmann, 2014; Kanzaki and Giblin-Davis, 2018). This could be partially because of the stability of habitat, i.e., newly dead wood environment, especially inner bark, does not last long, and the nematodes need to be transferred to appropriate habitat by specialized carrier. While the soil/litter habitat are rather consistent, i.e., leaf litter is constantly supplied. Thus, the nematodes are not necessary to be transported long distance to specific habitat, but can utilize wide-ranged arthropod as both carrier and substrate.

### Intraspecific variation in *P. degawai*

*Pristionchus degawai* is highly divergent in the mtCOI sequence, separated into 47 haplotypes, with 24, 15, and 8 types isolated from Mat, Nob, and both localities, respectively. The average number of haplotypes isolated from an individual millipede was 5.0 (SD 1.5; range 3-8) for Mat and 3.0 (1.2; 1-5) for Nob (Table 2), suggesting that the Mat population is genetically more divergent than the Nob population.

The haplotypes were separated into four groups according to the bootstrap values (Fig. 2B). Groups 1 and 2 were mostly found from Nob, group 3 was found from Mat, and group 4 consisted of strains from both localities. Considering the phylogenetic relationships (Fig. 2B), the Mat population could be more derived. However, to clarify the population structure, more materials from different localities are necessary. Compared with the first isolation, in which the nematodes were examined 1 week after the dissection, the species and genotypes were more divergent in the extensive survey, where the first-found individuals were cultured. In addition, *P*. *degawai*, which was found from all millipedes in the extensive survey, was not found in several millipedes in the first isolation (Tables 1 and 2). This suggests that one species/genotype dominates quickly (during 1 week of culture), and the nematodes should be examined in earlier cultures to evaluate diversity.

### Additional remarks

The primary purpose of this study was to establish many *Pristionchus* cultures to find undescribed species; so the sample size for the haplotype analysis was limited. However, regardless of the limitation, the haplotype diversity of *P. degawai*, 47 types, was much higher than expected. So far, population genetic studies of *Pristionchus* species have been conducted only on *P. pacificus* (Herrmann et al., 2010; Morgan et al., 2012, 2014), and no other species has been examined. *P. pacificus* is a hermaphroditic and cosmopolitan species. Thus, as a comparative system, similar analysis of more locally distributed gonochoristic species could be valuable. Further analysis of *P. degawai* and other gonochoristic species focusing on haplotype diversity (without establishing strains) will give more detailed population genetics information.

To date, several *Pristionchus* spp. have been isolated from millipedes, including *P*. *laevicollis*, *P*. *degawai*, *P*. *riukiariae* (Kanzaki et al., 2018), *P*. *arcanus* (Kanzaki et al., 2012a, 2012b), *P*. *pacificus* (Kanzaki et al., 2016, 2018), and several other species (Kanzaki, unpubl. data). *Pristionchus pacificus* is a hermaphroditic species with a cosmopolitan distribution that dominates in the lowland temperate zone in Japan (Herrmann et al., 2007; Kanzaki et al., 2016; Kanzaki, unpubl. obs.). Nevertheless, none of our more than 200 tentative strains was *P*. *pacificus*, suggesting that *Pristionchus* spp. segregate by altitude, temperature, or both.

Although many thelastomatid and rhigonematid gut parasites have been described from millipedes, phoretic/necromenic species have not been examined systematically, i.e., in addition to above species descriptions, Carta et al., (2018) isolated *Oscheius rugaoensis*, *Oscheius necromenus* (Sudhaus and Schulte, 1989) and *Mononchoides* sp. from *Chamberlinius hualienensis* collected at Hachijojima Island, Japan and Kanzaki et al. (2016) reported *P. pacificus*, *P. arcanus*, and *Oscheius* spp. from *Riukiaria* spp. collected in three localities in Japan (Fig. 3). In the present study, although detailed analysis was not conducted, an unidentified *Oscheius* sp. was associated with all examined millipedes. Considering the isolation records provided by Kanzaki et al. (2016) and Carta et al. (2018), the genus seems commonly associated with millipedes. *Oscheius* is a typical soil dwelling bacteria feeder, and all member of the genus are hermaphroditic species (Sudhaus, 2011), and presumed to be a competitor of *Pristionchus* spp. for the food (substrate for food bacteria). Further analysis of its diversity and ecology, e.g., whether they are competing or segregating, will give new insight to understand the biological interaction among *Pristionchus* spp. and other millipede associates.

In addition, *P*. *laminata* has other populations that do not undergo typical mass-occurrence and has several congeners in Japan (e.g., Tanabe, 2002). Further surveys of phoretic nematodes of millipedes, especially in cool mountain areas, will reveal further nematode diversity, including the satellite model group, *Pristionchus* spp. as well as other phoretic/necromenic species.
